# Essentially unedited deep‐learning‐based OARs are suitable for rigorous oropharyngeal and laryngeal cancer treatment planning

**DOI:** 10.1002/acm2.14202

**Published:** 2023-11-09

**Authors:** Jihye Koo, Jimmy Caudell, Kujtim Latifi, Eduardo G. Moros, Vladimir Feygelman

**Affiliations:** ^1^ Department of Radiation Oncology Moffitt Cancer Center Tampa Florida USA; ^2^ Department of Physics University of South Florida Tampa Florida USA

**Keywords:** deep learning OAR autosegmentation, head and neck treatment planning, optimization on autosegemented structures

## Abstract

Quality of organ at risk (OAR) autosegmentation is often judged by concordance metrics against the human‐generated gold standard. However, the ultimate goal is the ability to use unedited autosegmented OARs in treatment planning, while maintaining the plan quality. We tested this approach with head and neck (HN) OARs generated by a prototype deep‐learning (DL) model on patients previously treated for oropharyngeal and laryngeal cancer. Forty patients were selected, with all structures delineated by an experienced physician. For each patient, a set of 13 OARs were generated by the DL model. Each patient was re‐planned based on original targets and unedited DL‐produced OARs. The new dose distributions were then applied back to the manually delineated structures. The target coverage was evaluated with inhomogeneity index (II) and the relative volume of regret. For the OARs, Dice similarity coefficient (DSC) of areas under the DVH curves, individual DVH objectives, and composite continuous plan quality metric (PQM) were compared. The nearly identical primary target coverage for the original and re‐generated plans was achieved, with the same II and relative volume of regret values. The average DSC of the areas under the corresponding pairs of DVH curves was 0.97 ± 0.06. The number of critical DVH points which met the clinical objectives with the dose optimized on autosegmented structures but failed when evaluated on the manual ones was 5 of 896 (0.6%). The average OAR PQM score with the re‐planned dose distributions was essentially the same when evaluated either on the autosegmented or manual OARs. Thus, rigorous HN treatment planning is possible with OARs segmented by a prototype DL algorithm with minimal, if any, manual editing.

## 1 INTRODUCTION

Autosegmentation has the potential to accelerate pre‐treatment radiotherapy workflow and improve consistency, but autosegmented structures may often require lengthy manual adjustments, largely defeating the purpose.^1^, ^2^ While it was suggested that accurate delineation of salivary and swallowing structures may not be necessary for satisfactory Head and Neck (HN) treatment planning,^3^ the prevailing effort is clearly focused on improving the contours’ quality. With the introduction of deep learning (DL) technology in radiotherapy, the performance of autosegmentation algorithms has improved substantially.^4^, ^5^, ^6^, ^7^, ^8^, ^9^ A substantial number of papers, of which we will only cite the reviews^10^, ^11^, ^12^ and some recent efforts,^13^, ^14^, ^15^, ^16^, ^17^ were devoted specifically to autosegmentation of HN organs at risk (OARs). As a logical first step, quality of OAR autosegmentation is often judged by geometric concordance metrics against the human‐generated reference structures,^18^, ^19^, ^20^, ^21^, ^22^ sometimes followed by a *forward* dosimetric analysis, for example, comparing the dose‐volume histograms (DVHs) for the machine‐generated structures against the manual ones, based on the original dose distribution. However, the real goal of autosegmentation is the *inverse* ability: the quality of a plan generated on the (unedited) autosegmented structures should be maintained if the manual OARs are substituted for the automatic ones during the analysis. This approach is more labor intensive as it requires substantial replanning effort and subsequent rigorous plan quality assurance. As a result, only a small handful of publications employed the re‐optimization approach. Somewhat successful attempts included both atlas‐based^23^ and DL^24^, ^25^ methods, while deformable registration proved less promising.^26^ Van Rooj et al.^24^ used the automatic knowledge‐based planning system which streamlined the replanning effort but was never proven to produce treatment plans comparable in quality to highly experienced human operators, particularly in terms of the target dose homogeneity.^27^ Also, in these publications, dosimetric analysis was typically somewhat coarse, reporting maximum and mean OAR doses at most, without a detailed exploration of the DVH details. Hence, it is fair to say that to date, the literature is devoid of a definitive example of an automatic (DL) OAR segmentation method capable of producing structures usable for rigorous treatment planning without, or with minimal, editing.

A prototype DL autosegmentation algorithm trained on a consistent dataset of over 800 head and neck cases was recently introduced with promising results for the concordance and forward dosimetric metrics.^28^ In this work, we describe a rigorous evaluation of that algorithm for its ability to solve the inverse, and more important, problem: to generate OARs that when used for planning would produce dose distributions functionally similar in dosimetric quality to the original ones upon analysis on the reference structures.

## 2 METHODS

### 2.1 Data curation

The study sample size was estimated based on the assumption that the effect size can be approximated by the absolute dose‐differences for individual OARs corresponding to 5% change in normal tissue complicated probabilities (NTCP).^29^ In practice, extracting the exact effect values is somewhat complicated as the best current models are based on multivariable analyses.^30^, ^31^, ^32^ However, we only needed a rough estimate. Two NTCP models were used, one univariate for parotid glands^33^ and another two‐variable one for the swallowing dysfunction, with the dose to the pharyngeal constrictors as the primary variable.^31^ We started with an initial sample of 20 patients. From those data, the standard deviations (SD) of the means of paired differences were estimated. The smallest observable relative effect size (ratio between the dose‐difference causing a 5% NTCP change to the SD of the differences) was always above 1.0. Conservatively, a smaller but statistically “large” expected effect size of 0.8 was used instead. To achieve a power of 95% at the two‐sided level of significance of 5%, a minimum of 24 pairs would be required.

The sample size was increased to a total of forty previously treated head and neck cancer patients (26 base of tongue, 10 tonsil, 3 soft palate, and 1 supraglottic larynx, Table 1). These 40 patients were a separate cohort from the algorithm training or initial validation data. Patient data were anonymized under an IRB‐approved retrospective study protocol. The targets and OARs were originally delineated by a single experienced physician and treatment plans were generated using the RayStation treatment planning system (TPS) v. 9B or 11A (RaySearch Laboratories, Stockholm, Sweden) with the final dose calculated by a Monte Carlo algorithm. Primary and secondary PTV doses ranged from 60 to 70 Gy and 41.4 to 56 Gy, respectively. The plans were optimized for VMAT^34^ delivery (2‐4 arcs) on a TrueBeam series linear accelerator (Varian Medical Systems, Palo Alto, California, USA) equipped with a standard Millennium multi‐leaf collimator (5 mm leaf width in the center of the field). Deliverable dose distributions were calculated with 0.3% statistical uncertainty (1 SD for voxels above 50% of the maximum dose) on an isotropic 2.5 mm dose grid. For composite plans, primary and boost phases were planned separately, and summed dose clouds were used for comparative dosimetric evaluation.

**TABLE 1:** Patient population characteristics.

		Number	% or Range
Primary tumor site			
	Base of tongue	26	65.0
	Tonsil	10	25.0
	Soft palate	3	7.5
	Supraglottic larynx	1	2.5
Age			46‐81
p16/HPV+		33	82.5
Prescription dose (Gy)			
	70	10	25.0
	69.6	1	2.5
	68	1	2.5
	66	23	57.5
	60	5	12.5
Concurrent chemoradiotherapy		25	62.5
Prior treatment		1	2.5
T‐stage			
	0	0	0.0
	1	10	25.0
	2	15	37.5
	3	7	17.5
	4	8	20.0
N‐stage			
	0	2	5.0
	1	22	55.0
	2	12	30.0
	3	4	10.0
	4	0	0.0

### 2.2 Inverse comparison

For each patient, a set of 13 OARs was generated by the previously published DL‐based prototype auto‐segmentation model.^28^ For plan re‐optimization, the target contours were extracted from the original structure set and merged with the DL‐produced OARs. The following prescription constraints were in place for both original and re‐optimized plans: at least 95% of each planning target volume (PTV) received 100% of the prescribed dose (Rx) while the entire PTV volume minus 0.03 cc was covered by at least 95% of Rx. Additionally, the gross tumor volume (GTV) minus 0.03 cc received a full prescription dose. The maximum dose (to 0.03 cc) goal was 105% of the Rx, understanding that with the Monte Carlo dose engine the achievable result would more likely be in the 106−107% range.^27^ The newly replanned dose distributions were applied back to the manually delineated (reference) OAR structures. That created the following combinations of the dose distributions and structure sets: A—the original (clinical) dose evaluated on associated manual structures; B—the re‐optimized dose generated on the DL OARs and evaluated on those; and C—the re‐optimized dose distribution from B projected back on the original manual structures. Of those three options, the most pertinent comparison is between combinations B and C.

### 2.3 OAR geometric similarity and target dose conformality/homogeneity analysis

As a preliminary quality control tool, the geometric agreement between the original and the autosegmented OAR structures was evaluated with Dice similarity coefficient (DSC),^35^

(1) $$\mathrm{DSC}\;=\frac{2(V_{\mathrm{gt}}\;\cap \;V_{\mathrm{auto}})}{V_{\mathrm{gt}}+V_{auto}}\;$$

where $V_{\mathrm{gt}}$ and $V_{\mathrm{auto}}$ are volume of the ground truth and the autosegmented structures, respectively.

The primary PTV coverage was evaluated with inhomogeneity index II (Equation (2)),^36^, ^37^ and the relative volume of regret (the volume outside the primary PTV receiving ≥ R_x_ dose, relative to the PTV volume).^38^

(2) $$II\;=\frac{D_{max}-D_{min}}{D_{mean}}\;$$

where $D_{\max},\;D_{\min}$, and $D_{\mathrm{mean}}$ are the maximum, minimum, and mean dose to the PTV, respectively.

To assess the potential differences in primary target coverage between the original (A) and replanned (C) dose distributions, a paired two‐sided *t*‐test was performed using Graph Pad Prism software (v. 9, GraphPad Software, San Diego, California, USA). Since the target volumes were the same for all combinations, only one‐pair test was necessary. Here and elsewhere in this paper, a *p*‐value < 0.05 was considered statistically significant.

### 2.4 OAR dosimetric characteristics analysis

For the OARs, clinically important dose‐volume points were evaluated, as detailed in the Results section. A binary pass/fail score was assigned to each metric based on our planning objectives. The main question here was whether the re‐optimized dose distributions based on autosegmented structures (from B) would produce the same pass or fail results as the original one, when applied to the original manual structures (combination C). The numerical values of the DVH points were tabulated and the differences in mean values were analyzed between situations A, B, and C with a matched‐pairs ANOVA test. When the ANOVA *p*‐values indicated statically significant differences, the post‐hoc pairwise analysis was performed and *p*‐values for the B‐C comparison were reported. The same analysis was performed for the mean and maximum doses to each OAR.

In addition to the discrete points, the areas under the reference (re‐optimized dose distributions coupled with manual structures) DVH curves were compared by DSC with the corresponding areas under the comparison (re‐optimized dose with the DL‐produced structures) DVH curves using DVH data with 0.1 Gy resolution. This served as a proxy of similarity between the DVH shapes.

**FIGURE 1:**
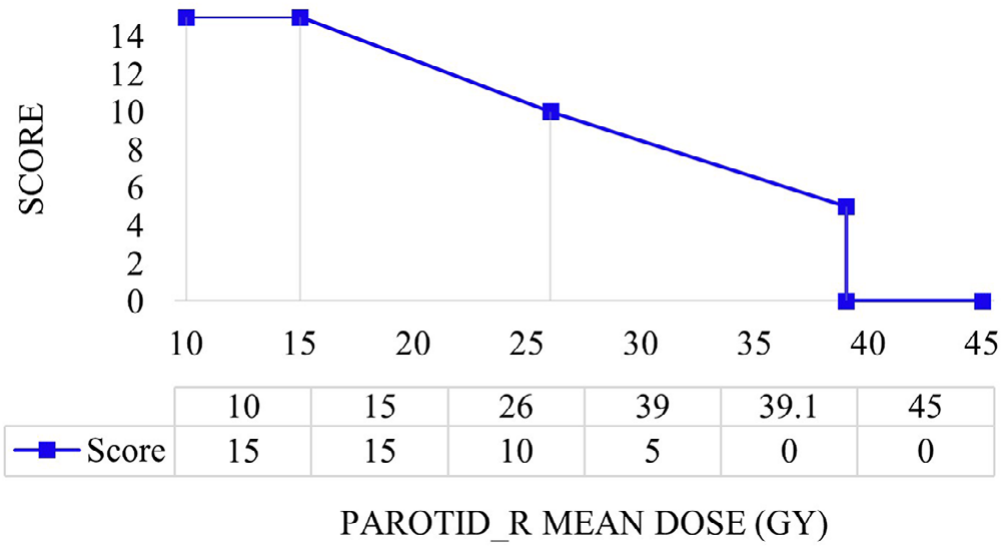
An example of the metric quality function. The clinical objective for the right parotid is the mean dose ≤ 39 Gy. The score functions are generally designed to have three regions; (a) ideal region: the maximum score is awarded, (b) transition region: the score increases gradually from zero to the maximum between the minimum acceptable and the ideal level of achievement, (c) failure region: minimum (zero) score is awarded.

Finally, to evaluate the overall quality of the re‐optimized dose distributions with the autosegmented and manual structures, a composite continuous plan quality metric (PQM, Equation 2) was calculated using PlanIQ software (Sun Nuclear Corp, Melbourne, Florida, USA).^39^ The advantages such approach were enumerated by Rayn et al.^40^ It was used for multi‐institutional plan challenges,^41^ evaluation of automated treatment planning tools,^39^, ^42^, ^43^ and is being integrated into some of commercial treatment planning systems.^39^, ^43^

(3) $$PQM\;=\sum w_{OAR}*f_{OAR}\left(x\right)\;$$

where $w_{OAR}$ is a weighting factor for each clinical objective and $f_{OAR}\left(x\right)$ is an individual linear score function. The continuous linear score functions (Figure 1) translated the degree of achievement of each clinical objective into numerical scores which are then combined into a total (composite) PQM score. As target coverage parameters were enforced prior to analysis, only the OAR objectives were reflected in the composite scores. While the relative importance of individual scores assigned to each OAR is necessarily subjective, if the same set is used for all cases the composite metric provides a consistent plan quality proxy for comparison. It is expected that the PQM scores would not approach 100 as with a few exceptions (e.g., the spinal cord), target coverage was prioritized over OAR sparing. Again, ANOVA analysis was performed to establish if there were statistically significant differences between the A, B, and C PQM scores.

## 3 RESULTS

The nearly identical primary target coverage for the original and re‐optimized plans was achieved, with essentially the same II and volume of regret values (Table 2). The difference in the mean PTV coverage between the original and re‐optimized treatment plans (A vs. B) was statistically insignificant in all four metrics.

**TABLE 2:** Mean values ± 1 SD for target coverage indices, with *p*‐values for *t*‐test of averages.

	Inhomogeneity index		Rel. volume of regret	
Original plan (A)	0.12 ± 0.03	*p* = 0.69	0.22 ± 0.10	*p* > 0.99
Re‐optimized plan (B)	0.12 ± 0.03		0.22 ± 0.13	

Same target volumes with dose distributions optimized on either manual or DL OARs.

In agreement with prior work,^28^ for the 13 HN OARs, sufficiently close geometric similarity between the manual and autosegmented structures was observed with an overall mean DSC value of 0.82 ± 0.08, which is comfortably above the typically considered cutoff threshold of the reasonable agreement between contours (DSC > 0.7).^44^

The overall mean values of the DSC of the areas under the corresponding pairs of DVH curves between A‐C, A‐B, and B‐C were 0.94 ± 0.11, 0.96 ± 0.06, and 0.97 ± 0.06, respectively (Table 3). The minimum value of 0.84 was recorded for the esophagus A‐B comparison. Focusing on B‐C column (replanned dose on original vs. autosegmented structures), except for the esophagus (0.9), the mean DSC values were at or above 0.95, indicating that the curves were largely indistinguishable.

**TABLE 3:** Mean values of DSC of areas under the DVH curves for each OAR.

OAR	DSC of areas under the DVH curves		
	A‐B	A‐C	B‐C
Bone_Mandible	0.97 ± 0.02	0.97 ± 0.01	0.99 ± 0.004
Brainstem_3 mm	0.95 ± 0.02	0.95 ± 0.02	0.96 ± 0.03
Cavity_Oral	0.95 ± 0.04	0.97 ± 0.02	0.95 ± 0.03
Cerebellum	0.95 ± 0.04	0.95 ± 0.04	0.99 ± 0.01
Esophagus_S	0.84 ± 0.16	0.91 ± 0.08	0.90 ± 0.07
Glnd_Submand_ipsi	0.95 ± 0.18	0.99 ± 0.01	0.98 ± 0.01
Glnd_Submand_contra	0.97 ± 0.02	0.97 ± 0.02	0.95 ± 0.18
Glnd_Thyroid	0.97 ± 0.02	0.98 ± 0.02	0.98 ± 0.02
Larynx	0.97 ± 0.01	0.98 ± 0.01	0.99 ± 0.01
Musc_Constrict	0.90 ± 0.26	0.96 ± 0.16	0.98 ± 0.01
Parotid_ipsi	0.96 ± 0.04	0.97 ± 0.02	0.97 ± 0.04
Parotid_contra	0.93 ± 0.08	0.94 ± 0.08	0.97 ± 0.02
SpinalCord_5 mm	0.94 ± 0.03	0.96 ± 0.02	0.97 ± 0.02

A: The original dose and (manual) structures, B: The re‐optimized dose on the DL‐produced OARs, and C: The re‐optimized dose distributions projected on the manual structures.

In addition to the shape analysis, Table 4 quantifies the mean and maximum dose to each OAR.

**TABLE 4:** Mean and maximum doses of OARs and *p*‐values (statistically significant in bold) from the ANOVA test.

		Average Dose ± 1SD (Gy)			*p*	
OAR	D_mean_ or D_max_	A	B	C	ANOVA	B‐C
Bone_Mandible	mean	25.1 ± 8.3	25.3 ± 8.6	24.9 ± 8.4	**0.045**	**0.038**
	max	62.4 ± 10.2	62.1 ± 9.4	63.1 ± 8.8	**0.009**	**0.007**
Brainstem_3 mm	mean	4.1 ± 1.7	4.1 ± 1.8	3.9 ± 1.7	**<0.0001**	**<0.0001**
	max	25.6 ± 7.5	25.3 ± 8.2	24.9 ± 8.0	0.343	**–**
Cavity_Oral	mean	27.0 ± 10.1	25.6 ± 10.1	27.6 ± 10.5	**<0.0001**	**<0.0001**
	max	69.2 ± 4.9	69.7 ± 3.7	69.9 ± 3.7	0.319	**–**
Cerebellum	mean	6.7 ± 3.3	6.8 ± 3.3	6.8 ± 3.3	0.655	**–**
	max	29.3 ± 7.2	29.5 ± 7.1	29.9 ± 6.9	0.186	**–**
Esophagus_S	mean	15.8 ± 7.8	16.1 ± 8.1	14.1 ± 7.5	0.564	**–**
	max	39.4 ± 11.3	36.4 ± 13.5	38.8 ± 11.3	**0.0003**	**0.003**
Glnd_Submand_ipsi	mean	58.5 ± 8.9	56.4 ± 9.9	55.9 ± 10.0	0.564	**–**
	max	61.1 ± 4.7	60.8 ± 3.2	61.4 ± 3.0	0.452	**–**
Glnd_Submand_contra	mean	33.1 ± 15.7	33.2 ± 15.6	32.5 ± 15.6	0.761	**–**
	max	59.8 ± 16.2	59.8 ± 16.3	60.2 ± 16.1	**0.042**	**0.043**
Glnd_Thyroid	mean	34.2 ± 12.0	34.4 ± 12.1	33.9 ± 12.1	0.175	**–**
	max	54.8 ± 12.0	54.5 ± 11.8	55.3 ± 11.6	**0.025**	**0.019**
Larynx	mean	33.5 ± 11.1	34.3 ± 11.8	34.3 ± 11.7	0.860	**–**
	max	65.2 ± 8.9	64.2 ± 9.4	65.7 ± 7.6	**0.025**	**0.021**
Musc_Constrict	mean	39.9 ± 8.9	41.1 ± 8.6	40.0 ± 8.5	**<0.0001**	**<0.0001**
	max	68.5 ± 4.7	69.0 ± 3.6	69.0 ± 3.6	0.379	**–**
Parotid_ipsi	mean	24.9 ± 10.8	23.7 ± 9.4	24.1 ± 9.2	0.384	**–**
	max	65.9 ± 7.8	66.4 ± 7.4	66.6 ± 7.2	0.442	**–**
Parotid_contra	mean	14.1 ± 8.6	13.7 ± 11.6	14.1 ± 11.9	0.059	**–**
	max	47.0 ± 18.1	47.4 ± 17.7	47.5 ± 18.1	0.427	–
SpinalCord_5 mm	mean	16.7 ± 3.9	15.9 ± 3.7	16.1 ± 3.7	**<0.0001**	0.1725
	max	36.9 ± 6.5	37.6 ± 5.9	36.5 ± 5.7	**0.003**	**0.002**

**FIGURE 2:**
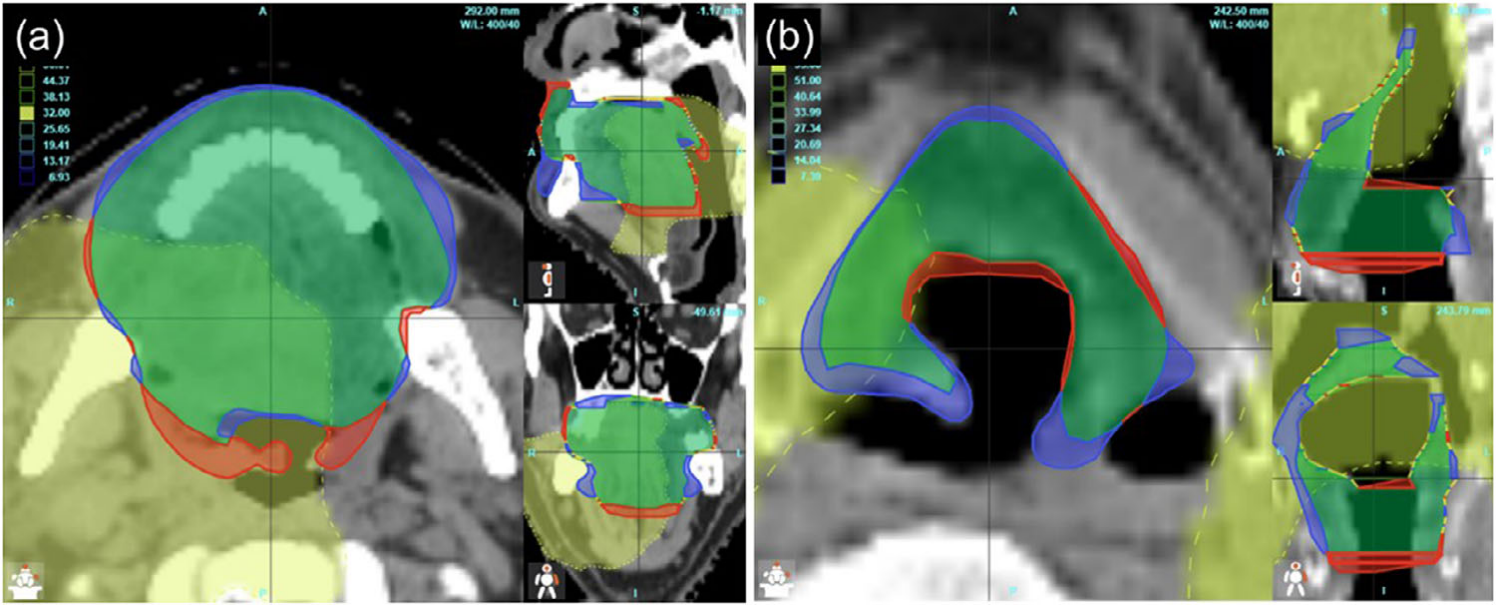
An example of the cases that met the clinical objective with the re‐planned dose and autosegmented structures but failed with the manual (reference) structures. Red: missing, blue: extra, green: common volume. (a) oral cavity structures with 32 Gy dose cloud, and (b) larynx structures with 55 Gy dose cloud.

The number of critical dose‐volume points which met the clinical objectives with the dose re‐optimized on the autosegmented structures but failed with the same dose distribution on the original manual structures was 5 of 896 (0.6%, Table 5).These included the larynx (2), oral cavity (1), submandibular gland (1), and pharyngeal constrictors (1). The pass‐or‐fail scores were often narrowly divided by the criteria falling in between close values. For example, in the larynx, the clinical objective was D_mean_ < 51 Gy and it was 49.4 Gy with the autosegmented structures and 51.1 Gy with the manual ones. However, in two cases, the dose differences were numerically more substantial. In one case, the mean doses to the oral cavity were 34.1  and 31.7 Gy with the manual and autosegmented structures, respectively, while the clinical goal was < 32 Gy. This was due to the difference in the definition of the oral cavity in inclusion/exclusion of the maxillary bone and overcontouring of the lip (Figure 2a). In this case, the volume of the manually drawn oral cavity was 181.1 cc while the volume of the autosegmented oral cavity was 249.0 cc. Despite meeting the objectives, because of the subtle differences in structure definition, the overall average mean dose to the autosegmented oral cavity from the re‐optimized dose distributions was lower than to the manually drawn structures: 25.6 ± 10.1 with autosegmentation versus 27.6 ± 10.5 Gy on the manual contours. For the larynx disagreement case, the volumes covered by 55 Gy were 34.5% and 30.3% for manual and autosegmented structures, respectively, with the clinical goal of < 32%. The autosegmented structure included more air, missed two slices at the cranial end in the high‐dose region and included extra two slices at the caudal edge in the low‐dose region (Figure 2b).

**TABLE 5:** Number of failures and mean values for OARs DVH objectives.

Objectives		Number of failures to meet objective			Metric Mean ± standard deviation			*p*	
OAR	Dose‐Volume Limit or Dmax or Dmean	A	B	C	A	B	C	ANOVA	B‐C
Bone_Mandible	V(70 Gy) ≤ 6.5%	1	1	1	0.5 ± 2.2	0.3 ± 1.3	0.3 ± 1.5	0.246	–
	V(60 Gy) ≤ 35%	1	1	1	3.4 ± 7.6	3.2 ± 7.4	3.3 ± 7.4	0.059	–
	V(50 Gy) ≤ 62%	0	0	0	7.5 ± 11.9	7.2 ± 12.2	7.3 ± 11.9	0.196	–
Brainstem_3 mm	Dmax < 54 Gy	0	1	0	25.5 ± 7.2	25.3 ± 8.2	24.9 ± 8.0	0.343	–
	Dmean ≤ 36 Gy	0	0	0	4.0 ± 1.6	4.1 ± 1.8	3.9 ± 1.7	<0.0001	<0.0001
Cavity_Oral	Dmean < 32 Gy	12	11	12	27.1 ± 10.1	25.6 ± 10.1	27.6 ± 10.5	<0.0001	<0.0001
Cerebellum	V(50 Gy) ALARA	–	–	–	0.0 ± 0.0	0.0 ± 0.0	0.0 ± 0.0	–	–
Esophagus_S	Dmean < 34 Gy	0	1	0	15.3 ± 7.7	16.1 ± 8.1	14.1 ± 7.5	0.564	–
Glnd_Submand_ipsi	Dmean < 39 Gy	25	25	25	56.2 ± 9.6	56.4 ± 9.9	55.9 ± 10.0	0.564	–
Glnd_Submand_contra	Dmean < 39 Gy	6	6	7	32.5 ± 15.5	32.2 ± 15.6	32.5 ± 15.6	0.761	–
Glnd_Thyroid	10‐15 cc: Dmean < 10 Gy	30	30	30	34.3 ± 12.0	34.4 ± 12.0	33.9 ± 12.1	0.175	–
	20 cc: Dmean < 25 Gy								
	25 cc: Dmean < 40 Gy								
Larynx	V(35 Gy) ≤ 79%	6	6	6	39.2 ± 31.0	38.3 ± 31.9	38.7 ± 31.8	0.614	–
∖	V(45 Gy) ≤ 45%	6	6	6	22.4 ± 23.3	22.4 ± 24.1	22.9 ± 24.4	0.552	–
	V(55 Gy) ≤ 32%	5	5	6	14.2 ± 16.9	14.2 ± 18.0	14.6 ± 18.0	0.640	–
	V(65 Gy) ≤ 22%	6	5	5	7.9 ± 12.0	8.0 ± 12.3	7.8 ± 12.1	0.824	–
	Dmean < 51 Gy	4	4	5	34.5 ± 11.3	34.2 ± 11.8	34.3 ± 11.7	0.860	–
Musc_Constrict	Dmean < 54 Gy	3	3	4	40.2 ± 8.7	41.3 ± 8.8	40.3 ± 8.9	<0.0001	<0.0001
Parotid_ipsi	Dmean < 26 Gy	7	8	8	23.6 ± 8.0	23.7 ± 9.4	24.1 ± 9.2	0.384	–
Parotid_contra	Dmean < 26 Gy	3	3	3	14.5 ± 11.5	13.7 ± 11.6	14.1 ± 11.9	0.059	–
SpinalCord_5 mm	Dmax < 50 Gy	0	0	0	36.6 ± 6.2	36.5 ± 5.7	35.8 ± 6.1	0.003	0.002
	Dmean ≤ 26 Gy	0	0	0	16.8 ± 3.9	15.9 ± 3.7	16.1 ± 3.7	< 0.0001	0.173
	V(30 Gy) ≤ 45%	3	3	2	15.4 ± 13.7	12.9 ± 13.0	12.8 ± 12.9	0.015	0.996
	V(40 Gy) ≤ 10%	1	1	1	0.6 ± 2.0	0.6 ± 2.4	0.5 ± 2.4	0.833	–

A: The original dose with the manual structures, B: Dose re‐planned and evaluated on DL‐produced OARs, and C: Re‐planned dose evaluated on the manual structures.

The autosegmented esophagus structures tends to be shorter in length than the manually drawn ones due to the model training specifics, resulting in an apparent statistically significant reported difference in the mean dose. However, after manually extending the autosegmented esophagus, this statistical significance disappears. For the rest of the structures, the absolute differences of the mean values are small (< 1 Gy for D‐ or < 1% for V‐parameters and as such are unlikely to meaningfully influence NTCPs.

The average OAR PQM scores were essentially identical: 68.9 ± 15.2, 69.2 ± 15.2, and 69.1 ± 15.4 for combinations A, B, and C, respectively. The ANOVA test did not indicate statistically significant differences.

## 4 DISCUSSION

For autosegmented structures to be truly clinically useful, dose distributions optimized on the unedited or minimally edited autosegmented OARs must result in similar quality treatment plans when evaluated with the reference structures. We call this the inverse comparison to distinguish from the forward one, where the original dose distribution optimized on the reference structures is coupled with the auto‐segmented OARs. When executed thoroughly, the inverse comparison is also the most robust measure of the autosegmentation quality. The concordance metrics need interpretation, for example, what numerical value of the DSC constitutes acceptable agreement, while the clinician's ratings of acceptable versus nonacceptable are necessarily subjective.

This is the first study that meticulously applied the inverse test to a reasonably large number (40) of HN cases where the autosegmentation was performed by the same model. That model is also quite consistent, as it was trained on ∼800 clinical cases segmented by one expert.^28^ We consider this consistent OAR contouring, believed to be anatomically correct, as the strength of the study, but it also raises the question of how the algorithm would perform if there was more interobserver variability. In addition to the algorithm characteristics, the training data congruence with the institution's expectations is of importance, as the same algorithm trained on the different data will produce somewhat varying results.^28^

The planning process was rigorous, resulting in the original and reoptimized dose distributions providing the same target coverage and primary PTV dose homogeneity. Of note, we use more stringent primary target dose homogeneity criteria (II) than typically published. We previously reported an average Homogeneity Index (as defined by ICRU‐83 based on D2 and D98^45^ of 5^27^ which is about one‐half of the values that typically can be inferred from the literature.^46^, ^47^, ^48^ The average values of composite OAR plan metrics, when the dose optimized on DL OARs was used in conjunction with either DL or manual structures, were practically the same. The OAR DVH shapes were essentially indistinguishable as well (Table 3). Additionally, a number of clinically relevant individual dose‐volume points were evaluated, which is a strength of the current study. At the same time, while the original clinical plans were by definition judged “acceptable” by the physician, the replanning ones were evaluated by the quantitative metrics but were not given a formal assessment by the physician. It may be considered a deficiency of the study as the published planning study guidelines assigns added value to plan evaluations by clinicians.^49^

**FIGURE 3:**
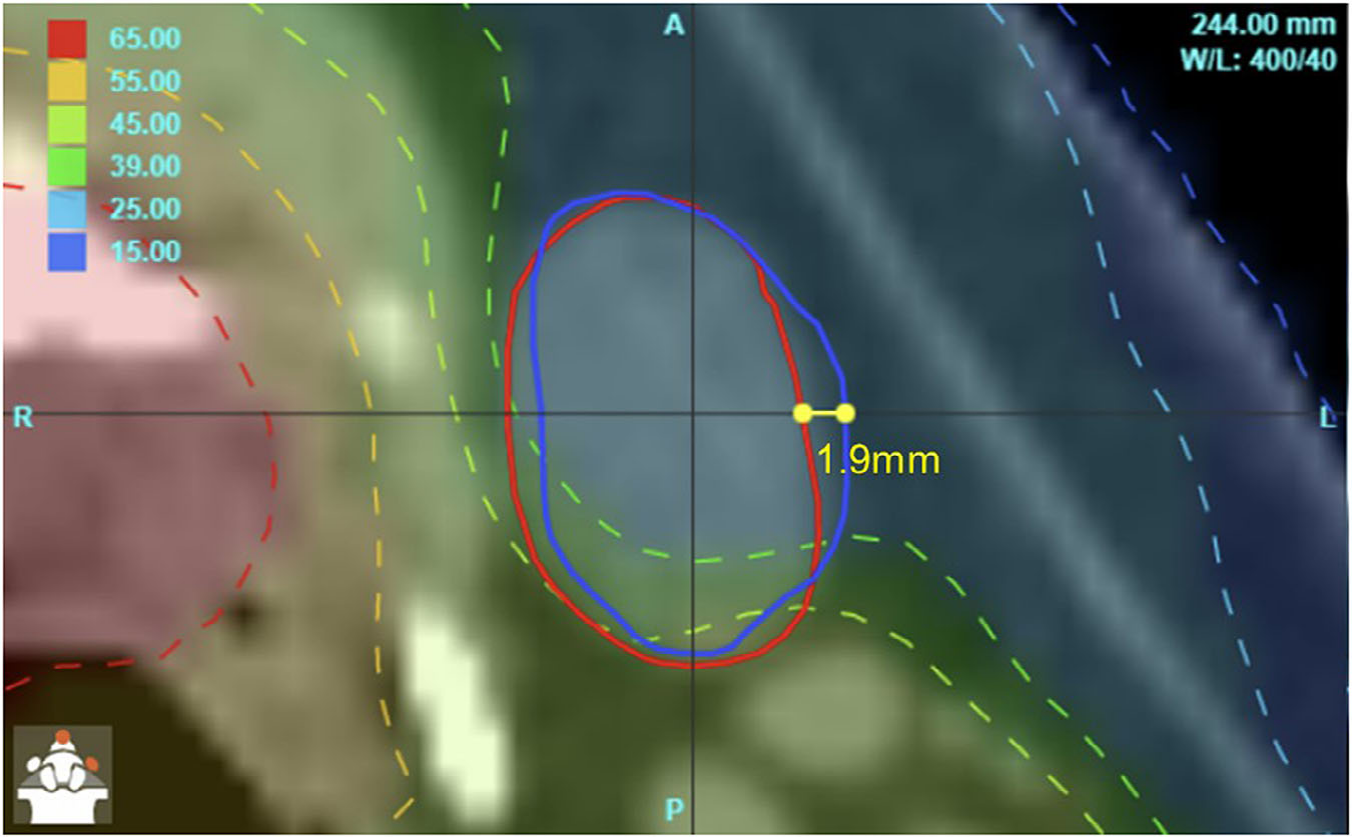
An example of a laterally shifted autosegmented submandibular gland (blue) compared to the manual structure (red).

The DL and reference‐structure based metrics derived from the reoptimized dose distributions passed or failed the objectives almost synchronously, with only 5 points found in disagreement (reoptimized dose passed on DL contours but failed on manual). Because of so few (5 of 896) cases of disagreement, it is difficult to discern any systematic pattern of failure of the DL algorithm. However, for larynx, it showed a tendency to contour one to two more caudal slides while missing one to two cranially, which can affect the mean dose to the structure depending on the target location. Also, in some of the submandibular glands, the DL‐produced structure was laterally shifted (< 2 mm), which influences the mean dose since the lateral dose gradient is typically present (Figure 3). Finally, the algorithm is known to poorly discriminate in the cranio‐caudal direction between the three pharyngeal constrictor muscles due to the low radiographic contrast. Thus, they were evaluated together previously^28^ and in this paper. If separate dose statistics are desired for the individual constrictor levels,^31^ they will have to be separated manually, which can be aided by a simple script.

After the definitive evaluation presented in this work, the following prudent practical workflow can be suggested once the prototype DL OAR segmentation algorithm is implemented in practice. After the simulation, the CT images should be sent directly to the autocontouring engine. Once the physician opens the plan to draw the targets afterwards, they also review the auto‐segmented normal structures, paying particular attention to the larynx, oral cavity, and perhaps submandibular glands. Hardly any edits should be required, however. After that, the case is ready for optimization. Of course, a reliable and fast DL autosegmentation algorithm would be particularly valuable for online adaptive HN therapy, given that dosimetric results with deformable registration were not necessarily encouraging.^26^

## 5 CONCLUSION

We have rigorously evaluated a prototype deep learning HN OAR autosegmentation algorithm for its ability to produce structures that when used for VMAT optimization result in dose distributions evaluating equally well on the original manual structure sets. On 40 cases, 896 individual DVH metrics were compared with a success rate of 99.4%. It is fair to say that in vast majority of cases the unedited DL‐generated OARs can be used for planning without compromising quality. The model also holds promise for online adaptive HN radiotherapy. However, despite encouraging results, this is still a single‐institution, single‐algorithm study. Before adopting a similar approach in clinical practice, it is necessary to validate the local algorithm/model performance and build confidence before routine use.

## AUTHOR CONTRIBUTIONS

Jihye Koo collected and analyzed the data, and drafted the manuscript. Jimmy Caudell contributed to the study design and interpretation of the results, and edited the manuscript. Kujtim Latifi and Eduardo G. Moros participated in the study design, provided critical feedback and contributed to the editing of the manuscript. Vladimir Feygelman designed the study, analyzed the data, and edited the manuscript. All authors reviewed and approved the final version of the manuscript.

## CONFLICT OF INTEREST STATEMENT

All authors were listed as investigators on and/or supported in part by the grant from Varian.
